# Changes in the Editorial Committee

**Published:** 1954-07

**Authors:** 


### CHANGES IN THE EDITORIAL COMMITTEE

Until he was elected President of the Society, Dr. Sutton had served us as treasurer both of the *Journal* and of the Society since 1946. He was also the senior member of the editorial committee and did much to keep the *Journal* alive in the last few years.

During the year we have also lost the services of Dr. Middlemiss, Dr. MacLachlan and Dr. Wood. The first two are now unable to spare the time. Dr. Middlemiss, in fact, served two months abroad as Adviser on radiology for the Colonial Office and his account of his journey is included in this issue. Dr. MacLachlan joined us three years ago and proved a stimulating and imaginative colleague. He now finds that the burdens of a city councillor and of a busy practitioner do not leave him time to do his editorial work to his satisfaction.

Dr. F. J. Y. Wood has gone to Southampton and we will miss him as editorial secretary very much.

